# Qualitative and quantitative analysis of chemicals emitted from the pheromone gland of individual *Heliothis subflexa* females

**DOI:** 10.1371/journal.pone.0202035

**Published:** 2018-08-14

**Authors:** Satoshi Nojima, Alice Classen, Astrid T. Groot, Coby Schal

**Affiliations:** 1 Department of Entomology and Plant pathology, North Carolina State University, Raleigh, North Carolina, United States of America; 2 Institute for Biodiversity and Ecosystem Dynamics, University of Amsterdam, Amsterdam, The Netherlands; 3 W.M. Keck Center for Behavioral Biology, North Carolina State University, Raleigh, North Carolina, United States of America; USDA Agricultural Research Service, UNITED STATES

## Abstract

The chemicals emitted from the sex pheromone gland of individual *Heliothis subflexa* females were sampled using a short section of thick-film megabore fused silica capillary column, and the pheromone glands of the same females were extracted after the effluvia collection. Both samples were treated with a silylation reagent, and then subjected to gas chromatography-chemical ionization-mass spectrometry for quantitative and qualitative analysis of all components. The total amount of all 11 components emitted from the glands of calling females was 153 ng/female/hr, which was substantially higher than previously reported. The ratios of the pheromone components in the volatile emissions and pheromone gland extracts were generally similar to previous studies, but with notable differences. The collections of volatiles and gland extractions contained, respectively: *Z*9-14:Ald (1.57%, 1.35%), 14:Ald (3.78%, 1.51%), *Z*7 + *Z*9-16:Ald (9.60%, 3.59%), *Z*11-16:Ald (76.14%, 18.94%), 16:Ald (2.95%, 2.17%), *Z*9-16:OH (0.07%, 7.21%), *Z*11-16:OH (1.11%, 49.04%), *Z*7-16:OAc (0.48%, 1.73%), *Z*9-16:OAc (1.32%, 4.02%), and *Z*11-16:OAc (2.98%, 10.43%). The thick-film megabore column is an efficient approach for sampling the headspace for semiochemicals.

## Introduction

In many insect species, volatile sex attractant pheromones play indispensable roles in the mating system, particularly in mate-finding [1]. The best characterized sex pheromones are in the Lepidoptera, an order of some 175,000 species, mostly moths, in which the pheromones of about 600 species have been identified [2]. In most moth species, sex pheromones are biosynthesized in a pheromone gland associated with the ovipositor in females, and pheromone components are emitted during a specific calling behavior [3]. The composition of the pheromone blend is species-specific, making it an important force in reproductive isolation and speciation [4].

Identification of the chemical structures and composition of pheromone blends is the foundation of understanding the chemical ecology and evolution of chemical communication. Most older pheromone identifications, and some contemporary studies, are based on gland extracts rather than emitted volatiles, mainly because the glands are easier to handle and extract, and relatively large amounts of candidate pheromone components can be obtained for analysis and bioassays. However, not all gland components serve as pheromone components because the pheromone gland may contain pheromone precursors, compounds that antagonize the attraction of heterospecifics, and related metabolic products that play no role in sexual attraction. Moreover, the ratio of compounds in gland extracts can be substantially different from their relative representation in the airborne pheromone, which is the actual chemical signal that the male perceives in the field [5, 6]. Therefore, it is essential to characterize the composition of the emitted pheromone in order to understand the dynamics and species-specificity of the pheromone communication system.

*Heliothis subflexa* (Guenée 1852) (Lepidoptera: Noctuidae) is a New World specialist that feeds only on species in the plant genus *Physalis* [7]. Its sex pheromone has been extensively investigated. Twelve aldehydes, acetate esters and alcohols that are biosynthetically related, were extracted from pheromone glands, including (*Z*)-11-hexadecenal (*Z*11-16:Ald) as the major sex pheromone component, and tetradecanal (14:Ald), (*Z*)-9-tetradecenal (*Z*9-14:Ald), hexadecanal (16:Ald), (*Z*)-7-hexadecenal (*Z*7-16:Ald), (*Z*)-9-hexadecenal (*Z*9-16:Ald), hexadecyl acetate (16:OAc), (*Z*)-7-hexadecenyl acetate (*Z*7-16:OAc), (*Z*)-9-hexadecenyl acetate (*Z*9-16:OAc), (*Z*)-11-hexadecenyl acetate (*Z*11-16:OAc), (*Z*)-9-hexadecen-1-ol (*Z*9-16:OH), and (*Z*)-11-hexadecen-1-ol (*Z*11-16:OH) [8, 9].

To assess which compounds function in intraspecific communication, it is essential to determine (a) which compounds are emitted from the pheromone gland during calling behavior, and (b) which of these contribute to the male’s mate finding process. Heath et al. [10] quantified pheromone gland components in collections of volatiles from individual *H*. *subflexa* females over a 2 hr period, and detected eight components (16:Ald, *Z*9-16:Ald, *Z*11-16:Ald, *Z*7-16:OAc, *Z*9-16:OAc, *Z*11-16:OAc, *Z*9-16:OH, and *Z*11-16:OH), with total release rates of 0.25 ng/min; *Z*11-16:OH represented 3.6% of the emitted volatiles. Although the amount of *Z*11-16:OH was small, it is a behaviorally significant component that plays an important role in intraspecific interactions [11–13]. In addition to these eight compounds, Lievers and Groot [14] also detected 14:Ald and *Z*9-14:Ald in the collections volatiles from *H*. *subflexa* females.

The early results guided flight tunnel and field trapping experiments that evaluated the effectiveness of various gland compounds as pheromone components. While flight tunnel experiments have been informative in delineating pheromone blends, results often conflict with trapping studies in the field. For example, flight tunnel experiments with *H*. *subflexa* concluded that *Z*11-16:Ald, *Z*9-16:Ald, and *Z*11-16:OH (applied to filter paper) were essential components for attracting males, but *Z*11-16:OAc did not contribute to male attraction [13]. Trapping studies, however, demonstrated that trap catch increased when the three acetates were added to either a binary blend of *Z*11-16:Ald and *Z*9-16:Ald (dispensed from a polyethylene vial) [8] or a 4-component blend that also included 16:Ald and *Z*11-16:OH (dispensed from rubber septa) [11]. *Z*7-16:OAc and *Z*11-16:OAc were particularly effective in increasing trap catches in both North Carolina and Mexico [11]. Likewise, in flight tunnel assays *Z*11-16:OH significantly elevated male responses over a wide range of ratios– 1% to 50% relative to *Z*11-16:Ald [13], whereas trap catch in the field was elevated only at low amounts of *Z*11-16:OH (5.8% of *Z*11-16:Ald loaded on rubber septa), and amounts >11% depressed trap catch [8, 12]. Whether these disparities were due to different dispensers or different expression of behavior in the wind tunnel and the field remains to be determined. Nevertheless, the latter results were generally confirmed in trapping studies in North Carolina and Mexico [11]. All studies to date have discounted any behavioral role for 14-carbon aldehydes [11].

We were particularly motivated to investigate a new approach to collecting volatiles from calling females because (a) investigations of the involvement of *Z*11-16:OH in mate-finding in various *Heliothis* species have resulted in conflicting reports about its function, (b) differences were found among populations in studies involving blend ratios of *Z*11-16:OH [11], and (c) *Z*11-16:OH can be challenging to quantify in collections of volatiles. Most collection systems for volatile pheromones force air over a calling female, a group of females, or a synthetic pheromone source, and entrain the volatiles onto an adsorbent such as glass, SuperQ, Tenax, or activated charcoal [15–17]. Major challenges for these systems are contaminants associated with the adsorbents and the relatively large amounts of solvent required for the quantitative recovery of all analytes. Moreover, alcohols readily bind to active sites on glass and may be difficult to desorb. Sections of thick-film megabore capillary columns efficiently trap volatiles in preparative gas chromatography (GC) either at ambient temperature or with cooling [18, 19] and this approach was subsequently adapted to collect volatiles from synthetic sources of pheromones and individual calling females [20, 21]. Here, we apply this approach for trapping the volatile pheromone of *H*. *subflexa*. To detect lower amounts of alcohols, we also derivatized the collected samples and analyzed them by GC-CI-SIM-MS. We report the results of methods development and validation using synthetic pheromone components and quantify the emission of individual calling *H*. *subflexa* females.

## Methods

### Ethics statement

*Heliothis subflexa* moths were reared at North Carolina State University’s insectary. The use of these insects is exempt from approval from the Institutional Animal Care and Use Committee.

### Insects

*Heliothis subflexa* larvae were collected on *Physalis angulata* in 2005 in Clayton, North Carolina (35^o^39’58”N, 78^o^30’36”W), and reared on artificial diet supplemented with *P*. *angulata* fruits (tomatillo). The pupae were separated by sex and maintained in a group in an acrylic cage (30 x 30 x 30 cm) at 24 ± 1°C, ~60% relative humidity, on a 14:10 light:dark cycle, with lights-off at 0500 hrs. Newly eclosed adults were collected daily and aged. We used 1–6 d old virgin females, at an average age of 4.1 d, as in previous studies [8, 10].

### Chemicals

Pheromone gland components and related chemicals were obtained from Pherobank (Wageningen, The Netherlands), Shin-Etsu Chemical (Tokyo, Japan) and Bedoukian Research (Danbury, CT, USA). Purity was generally >98% by GC, but Z9-16:Ald and Z11-16:OAc were >95% pure.

### Collection of volatiles

The collection procedure was developed and validated with authentic aldehydes, acetate esters and alcohols, and then with *H*. *subflexa* females based on techniques reported previously [18, 19]. The adsorbent trap consisted of a 20 cm section of thick film DB-1 column (0.53 mm ID, film thickness 5 μm) connected to a vacuum pump that delivered a flow rate of 5–15 ml/min. The connector was a stainless reducing union (3.2 mm OD to 1.6 mm OD) with a graphite ferule (Alltech Associates, Deerfield, IL, USA). To assess breakthrough, three 20 cm megabore traps were connected in series using a glass press fit connector (Alltech Associates). Column sections were checked for even cuts because an uneven cut can introduce reactive surfaces that diminish trapping efficiency. The collection traps were rinsed twice with 100 μl of methylene chloride (pesticide analysis grade) using a HPLC syringe connected to the trap with a glass press fit connector [18] and dried at room temperature over-night. For reuse, the traps were reconditioned by rinsing twice with 100 μl of methylene chloride, and the trap was carefully examined for square and even ends for a secure connection.

**Fig 1:**
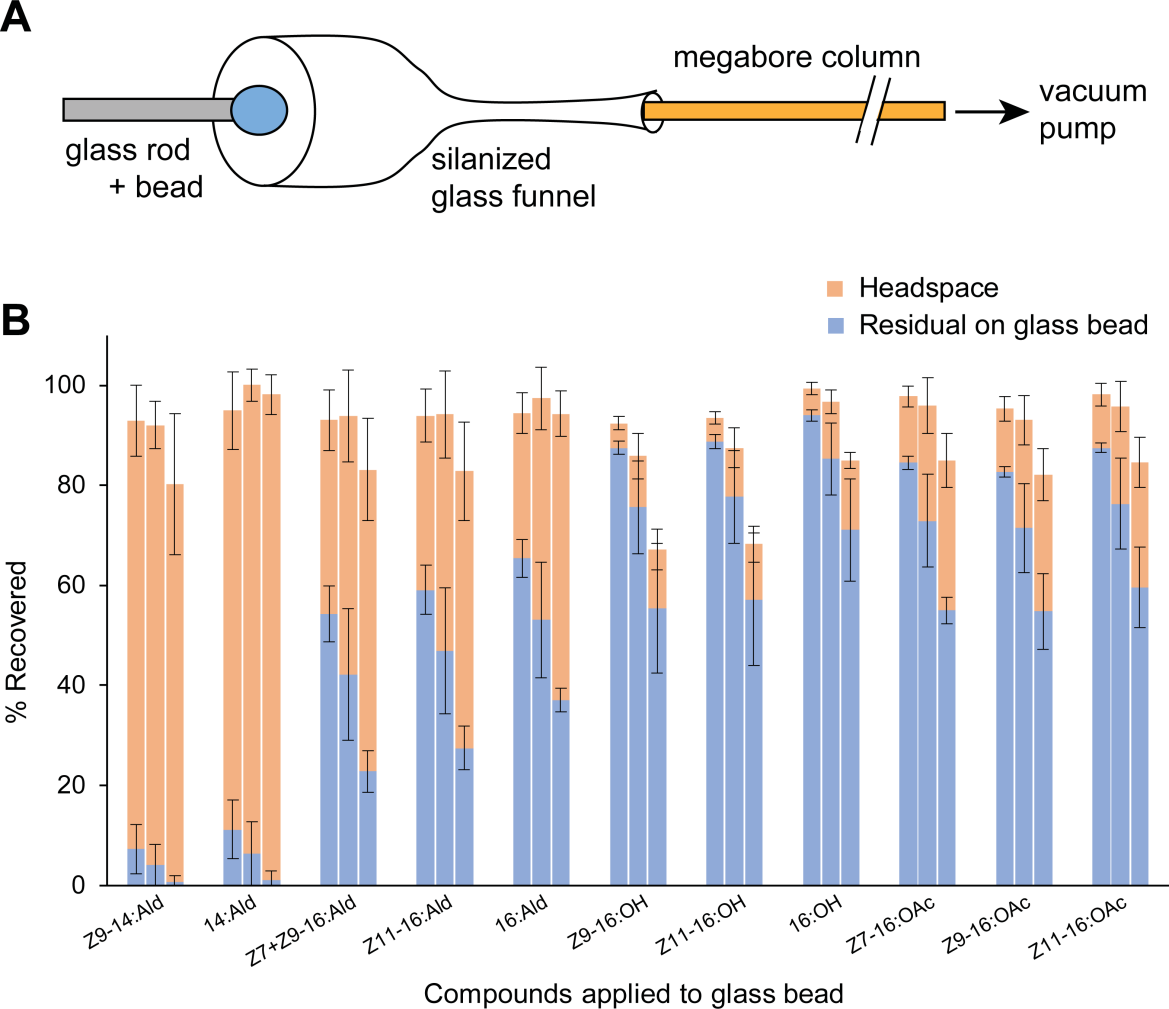
Volatilization of synthetic pheromone components from a glass bead representing an artificial pheromone gland. (**A**) Schematic diagram of the glass dummy and collection apparatus used to trap volatile pheromone components. A 20 cm megabore column was used. (**B**) Comparison of residual compounds extracted from the glass bead after a 60 min collection session and the collections of volatiles (headspace) recovered from the megabore trap. Adjacent stacked bars within the same compound, from left to right, represent air flow rates of 5, 10 and 15 ml/min. Variation is SEM. *N* = 5 pairs of glass beads and collections of volatiles for each flow rate.

A glass funnel was made from a Pasteur pipet cut ~10 mm before the tapering point. The cut end was polished over a flame, and the other side cut to 10–20 mm and narrowed so that the megabore trap would fit in its inner diameter (Fig 1A). The funnel was deactivated with 5% dimethylchlorosilane in toluene (Glass Prep; Alltech Associates, Inc., Deerfield, Illinois), and attached to the upwind end of the megabore trap. The funnel was carefully positioned over a glass bead or the ovipositor of a calling female so that they were positioned inside the funnel and volatiles were collected from the ambient air. Control collections of ambient air were made and treated in the same manner as sample collections. Background values from control collections were subtracted from the collections from the glass bead or calling females.

A glass bead represented an artificial pheromone gland (Fig 1A). We used a glass rod (2 mm OD and 150 mm long), the tip of which was rounded over a flame and then abraded with #1000 abrasive paper. A solution of standard compounds containing 10 ng of each in 2 μl hexane was applied to the glass bead with a micro-capillary (Drummond, Sigma, St. Louis, MO, USA). After the solvent evaporated (~1 min), the glass rod was placed on a 30°C heating block to facilitate evaporation of compounds. Volatiles emanating from the bead were collected for 60 min using the megabore column system. After collection, the megabore trap was positioned vertically on a lab stand with the funnel upward [18, 19], and the other end was positioned over a 300 μl conical glass insert (Agilent). Then, a GC syringe was used to rinse the inside wall of the funnel with 10 μl hexane containing 20 ng internal standard (pentadecyl acetate, 15:OAc). The solvent went into the trap and extracted volatiles collected in the trap. The funnel and the megabore trap were then rinsed with two aliquots of 10 μl clean hexane. A glass press fit connector was attached to the upper end of the trap to recover the collected volatiles, when a funnel was not attached to the trap. The trap eluants were stored in 1.5 mL autoinjection vials in a -30°C freezer until use. The glass bead was immediately extracted in the same manner, and the extracts were combined in a 300 μl conical glass insert and stored in a 1.5 mL autoinjection vial.

Collections of volatiles from females were made between 3 and 6 h after lights-off, the peak calling period when maximal pheromone emission occurs in *H*. *subflexa* [10]. When collecting volatiles from a calling female, the vacuum pump was turned off when the pheromone gland was retracted, and turned back on when calling resumed, for 30 min of total calling time. Although we did not record the total assay time for each female, interruptions were minimal, and the 30 min collection was usually accomplished within 45 min. Background control collections were carried out in a cage of 5 virgin calling females. The funnel of the megabore trap was positioned in the center of the cage and the ambient air in the cage was collected for 30 min. This represented an extreme case to show that this collection method is highly directional, so we expected no significant amount of pheromone in these background collections. Sample processing was identical to collections of volatiles from glass beads, but the samples were treated with silyl agents and subjected to GC-MS analysis.

### Pheromone gland extraction

Collections of volatiles were made from 2 to 4 females on any given day, with a collection from only one calling female conducted at a time, because each female required careful attention to her calling status and re-orientation of the funnel relative to the pheromone gland. Heath et al. [10] showed that the pheromone gland content of *H*. *subflexa* declined between 6 and 8 hrs of the scotophase. Therefore, when collections from all females on a given day were completed (about 6 hrs into the scotophase), the pheromone gland of each female was dissected and extracted for 15 min in 50 μl of hexane containing 100 ng of 15:OAc as an internal standard in 300 μl conical glass insert. The conical vials were stored in 1.5 ml autoinjection vials in a -30°C freezer.

### Micro-chemical derivatization

Each sample was reduced to 10 μl under a gentle stream of high purity N_2_. To derivatize the alcohols, we added 3 μl of 1% BSTFA (*N*,*O*-Bis(trimethylsilyl)trifluoroacetamide) with 10% TMCS (trimethylchlorosilane) (Pierce, Rockford, IL, USA) in hexane. The sample was mixed and briefly centrifuged to draw the solution to the bottom of the vial. The reaction mixture was then incubated for 30 min at room temperature, reduced to 2–3 μl with N_2_, the inside wall of the vial was washed with 5 μl hexane, reduced to 2–3 μl, and the reaction mixture at the bottom of the conical vial was injected into the GC-MS. This procedure adds a trimethylsilyl (TMS) functional group to alcohols.

### GC-FID and GC-MS analysis

Sample analysis was conducted on a 7890 GC-FID equipped with a nonpolar EC-5 capillary column (30 m, 0.25 mm ID, 0.25 μm film thickness; Alltech Associates). For this analysis, alcohols were not derivatized. Helium was used as the carrier gas at a head pressure of 115 kPa (flow rate, 1.5 ml/min). Oven temperature was set at 50°C for 2 min, increased at 15°C/min to 250°C, and held for 5 min. The injector and detector temperatures were set at 270°C. Septum purge flow rate was set at 3 ml/min with a total flow rate of 14 ml/min in the split injection mode (split ratio of 1:10), whereas the total flow rate was set at 50 ml/min in the splitless injection mode with the purge valve off for 1 min. A dilution series of standard chemicals was used for calibration of target compounds.

We used a 6890 GC (Agilent) coupled to a 5975 MSD (Agilent) and equipped with a DB-5ms column (30 m x 0.25 mm x 0.25 μm, Agilent) with a 2 m retention gap as a guard column. Injector temperature was 270°C and samples were introduced in pulsed splitless mode (20 psi for 2 min, then 9.4 psi). Helium was the carrier gas at a constant flow of 1.2 ml/min. The oven was programmed from 50°C (2 min hold) to 210°C at 4°C/min and then to 260°C at 20°C/min (10 min hold). MS quadrupole temperature was 150°C, MS source temperature was 230°C, and transfer line temperature was 250°C. The MS was operated in chemical ionization mode with methane as reagent gas. Fragmentation patterns of standard chemicals were analyzed with GC-CI-MS in Scan mode. Non-target compounds in the effluvia collection and extracts which eluted close to target compounds were also examined with GC-CI-MS in Scan mode, then characteristic fragments for SIM analysis of pheromone compounds were selected. We monitored the following ions by SIM (*m*/*z*): *Z*9-14:Ald (209, 211, 239), 14:Ald (211, 213, 241), *Z*7-16:Ald (237, 239, 267), *Z*9-16:Ald (237, 239, 267), *Z*11-16:Ald (237, 239, 267), 16:Ald (239, 241, 269), 15:OAc (internal standard, 269, 271, 311), *Z*9-16:OH(TMS) (297, 311), *Z*11-16:OH(TMS) (297, 311), 16:OH(TMS) (299, 315), *Z*7-16:OAc (221, 222, 283), *Z*9-16:OAc (221, 222, 283), and *Z*11-16:OAc (221, 222, 283). These fragments were monitored only at specific time windows in which each target compound eluted from the column to increase sensitivity and quantitative capability. A dilution series of standard chemicals was also used for calibration of target compounds.

### Statistical analysis

The paired Student’s *t*-test was used (α = 0.05) for comparisons of glass bead or gland extracts and the respective collection of volatiles from the same bead or female, respectively.

## Results and discussion

### Methods development and validation

We tested the effects of collecting odors with and without a silanized glass funnel (Fig 1A and S1 Table), by various segments of 20 cm column sections connected in series, and at various air flow rates. We assessed the efficiency of trapping various authentic aldehydes, acetate esters and alcohols. Without the silanized glass funnel, only negligible amounts of volatiles were trapped (data not shown), consistent with previous observations even in a closed system that the megabore trap rapidly lost its capability to collect odors away from the sources [20]. In an open system, when air can enter the capillary column from all directions, its position relative to the pheromone source is critical. Likewise, we detected no or only negligible amounts of pheromone components from a cage of five virgin females with the collection trap positioned in the center of the cage. These represented background collections and demonstrated the directionality of the trap and a requirement that it be near the pheromone source. Given the small diameter of the collection trap (0.53 mm), relatively low flow rates must be used, compared to conventional adsorbent traps. At low flow rates, the active air space sampled by the opening of the trap is very small, and compounds emanating from the glass bead volatilize in all directions and most are not trapped. The use of the funnel limited the sampling volume to the vicinity of the glass bead and directed airflow over the bead and toward the trap. Moreover, the funnel enabled some flexibility in placing the megabore column trap near the pheromone gland of a calling female. Similar findings were reported in collections from septa as pheromone sources–the relative position of the pheromone source and the trap intake significantly affected the quantity of volatiles recovered [20]. In addition, the funnel mitigated the flexibility of the megabore column which made it difficult to position the column near the pheromone gland.

We also compared the integrity and trapping efficiency of these compounds with air and helium flow at 10 ml/min (considered optimal from other experiments, below) over the glass bead to confirm that no hydrolysis of compounds occurred with air flow. We found negligible amounts of the standard chemicals, and most often below our detection limits, in the second and third sections using 10 ng each of the standard chemicals. Thus no significant breakthrough occurred from the first trap (data not shown). We tried longer 40 cm traps to add a margin of safety. While they worked well with the glass bead, we found them to be impractical because the added flexibility of the column made it difficult to position the funnel near the female’s ovipositor as the female changed her position. A 20 cm section is stiffer, and much easier to handle, so it was used for all collections of volatiles from calling females.

The linear velocity through a 0.53 cm ID column at ambient temperature would vary from ~35 cm/sec at 5 ml/min to ~115 cm/sec at 15 ml/min. We anticipated a tradeoff between low and high flow rates. Low flow rates might fail to direct all the volatiles into the megabore column trap; at the same time however, low flow rates should favor greater interactions of volatiles that enter the trap with the stationary phase of the megabore column and thus increase trapping efficiency. Conversely, very high linear velocities over the glass bead should direct more pheromone into the trap, but trapping efficiency may be compromised because of less interaction between the analyte and the stationary phase.

Using a silanized funnel and a 20 cm megabore column trap, we investigated the trapping efficiency of this system at three air flow rates using a glass bead as an artificial dummy representing the pheromone gland. A blend of typical pheromone components including aldehydes, acetates, and alcohols was applied to the glass bead, and we collected volatiles using the megabore column. The compounds remaining on the bead were immediately extracted after the collection of volatiles. Both sets of samples were quantified using GC-FID.

The total recovery of each component, which included components trapped as volatiles and extracted from the beads, ranged from 67% to 100%, with a strong influence of flow rate (Fig 1B). The total recovery of compounds ranged from 92% to 99% at 5 ml/min and from 67% to 98% at 15 ml/min. For all compounds, higher flow rates resulted in greater emissions, as indicated also by less mass remaining on the glass bead. For most compounds, the recovery also varied with air flow rates–high flow rates resulted in lower total recoveries of compounds, likely because of lower trapping efficiency of the megabore column. There was also a clear pattern of recovery of volatiles vs. residual compounds extracted from the glass bead. Considering only the intermediate flow rate of 10 ml/min, 94–95% of the two 14-carbon aldehydes were emitted and only 5–6% remained on the glass bead, whereas for the three 16-carbon aldehydes 46–55% were emitted and 45–54% remained on the glass bead (Fig 1B). The emitted three 16-carbon acetates ranged from 20% to 24% (76–80% remained on the glass bead), and only 11–12% of the three alcohols were emitted while the rest (~90%) remained on the glass bead.

These results indicate that the evaporation of compounds from a substrate depends on the air flow rate and the physical and chemical properties of each component, such as its functional groups, chain length, volatility and polarity. Nevertheless, this collection system effectively and quantitatively trapped compounds that evaporated from the surface of the glass bead. An air flow rate of 10 ml/min, generating a linear velocity of ~75 cm/sec represents a compromise between a high flow rate that directs more volatiles into the trap, and lower trapping efficiency due to reduced interaction of volatiles with the stationary phase at high flow rates. Low flow rates (eg, 5 ml/min) result in high total recovery of compounds, but a higher fraction of the compounds was recovered from the substrate to which they were applied; that is, they were not volatilized. Since we were particularly interested in recovering alcohols, low flow rates would be disadvantageous.

Overall, at 10 ml/min, the representation of compounds in the trapped volatiles differed dramatically from the loaded blend. We loaded 10 ng of each of the 12 compounds on the glass bead. We recovered the following percentages relative to the total amount recovered from the megabore trap: *Z*9-14:Ald (18.6%), 14:Ald (19.8%), *Z*7 + *Z*9-16:Ald (21.9%), *Z*11-16:Ald (10.0%), 16:Ald (9.4%), *Z*9-16:OH (2.2%), *Z*11-16:OH (2.1%), 16:OH (2.4%), *Z*7-16:OAc (4.9%), *Z*9-16:OAc (4.6%), and *Z*11-16:OAc (4.1%).

Using a similar system, Kuenen and Hicks [20] demonstrated no breakthrough even with 1 hr collections at air flows as high as 25 ml/min. It is important to note, however, that they used a 75 cm column, and at high air flows analytes where recovered up to 65 cm from the proximal end of the column. At a flow rate of 10 ml/min, they recovered analytes within 20 cm of the column inlet in 1 hr collections. Also, Kuenen and Hicks [20] used a flow-through closed system that directed volatiles toward the trap opening, making a funnel unnecessary.

### Collections of volatiles from *H*. *subflexa* females

The pheromone gland volatiles of female *H*. *subflexa* were individually sampled using a silanized glass funnel, a 20 cm section of megabore column, and an air flow rate of 10 ml/min. After the collection, we dissected and extracted the pheromone gland of the same female. Both samples were treated with a silylation reagent, and then analyzed by GC-CI-MS.

**Fig 2:**
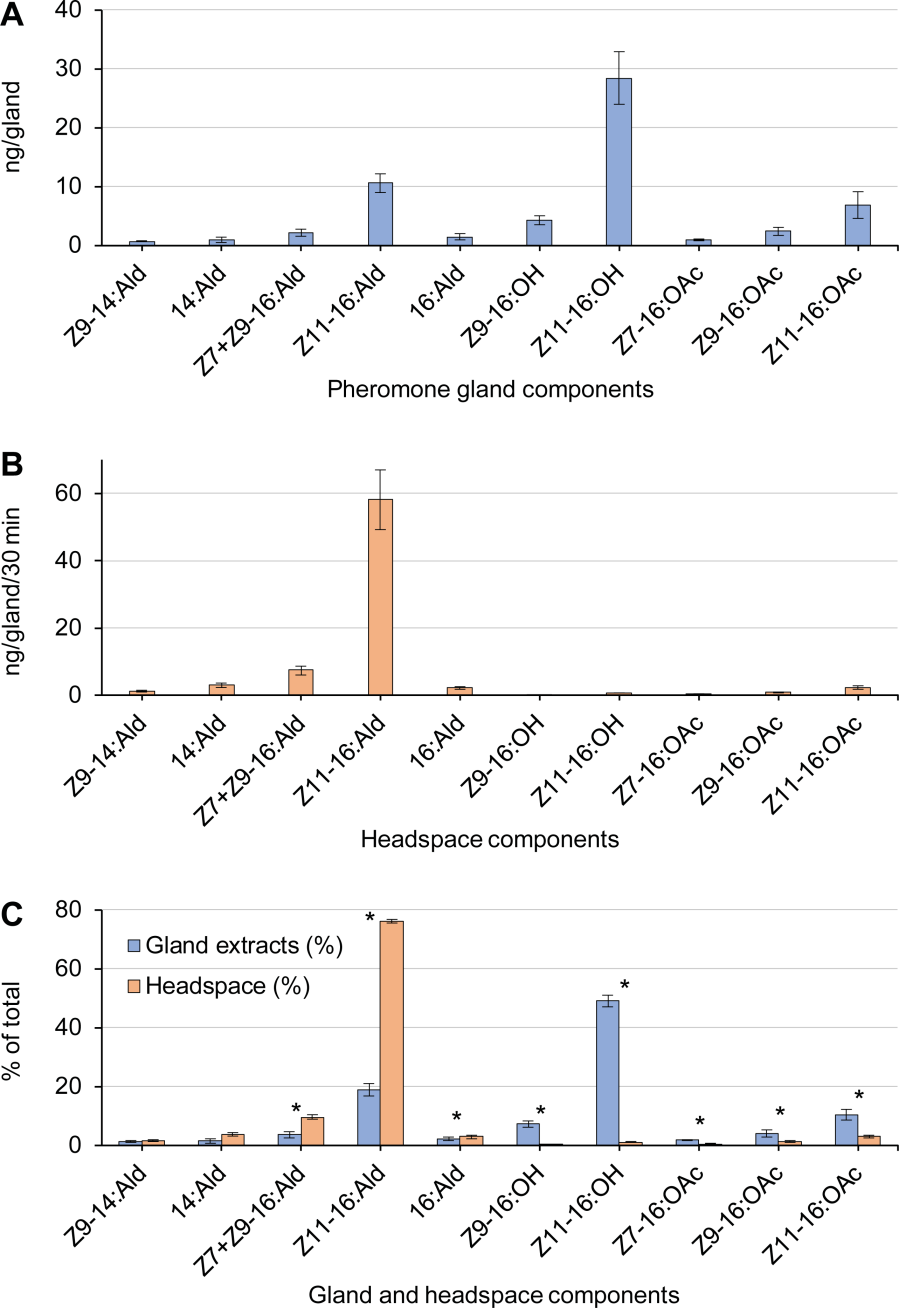
Collection of volatiles (headspace) and pheromone gland extracts of individual calling *Heliothis subflexa* females. (**A**) Emission rate (ng/30 min/female) of each pheromone component, *N* = 7. (**B**) Mass (ng) of each component extracted from the pheromone gland after the collection of volatile was completed, *N* = 7. (**C**) Representation of each component in the collections of volatile and gland extracts as a percentage of the respective total. Variation is SEM. * indicates significant difference (paired Student’s *t*-test, *P* < 0.05).

We extracted 58.2 ng from the pheromone gland after collection of volatiles, with *Z*11-16:OH and *Z*11-16:Ald most represented at 28.4 and 10.6 ng, respectively (Fig 2A and S1 Table). This was substantially less than in previous studies (eg, 333 ng in Heath et al. [10]; 245 ng in Lievers and Groot [14] after collections of volatiles). These differences could be related to the use of different strains, at different times during the night, and different extraction procedures. For example, *H*. *subflexa* females from St. Croix contained 738 ng of total pheromone components, whereas Florida females contained 333 ng [10]. Heath et al. [10] also showed that the pheromone content of *H*. *subflexa* from Florida rose from only 14 ng at the start of the scotophase to 333 ng 6 hrs later, and declined to 13 ng at the end of the scotophase. In our assays, successive collections of volatiles were made from individual calling females to assure that each female was calling and to re-orient the funnel relative to her pheromone gland. The pheromone glands were then extracted after all collections of volatiles were completed. It is possible therefore that the low recovery of pheromone in our gland extractions was related to the fact that glands were extracted later in the scotophase, when the pheromone titer in the gland was low.

The total of all volatile compounds collected from calling females was 76.4 ng/30 min per female, with *Z*11-16:Ald most represented at 58.2 ng (Fig 2B). This is substantially higher than previously reported. Heath et al. [10] recovered 30.6 ng total pheromone, and 16.5 ng of *Z*11-16:Ald per hr of collection at the peak of *H*. *subflexa* calling period [10]. Similarly, Lievers and Groot [14] recovered approximately 11.3–17 ng of total pheromone per hr per female. Therefore, the megabore column collection system collected ~5–13 times more volatile emissions from a single female than other studies that used polymeric adsorbents. Higher release rates were likely due to two factors: (a) Greater efficiency of collection, less solvent evaporation, and hence a more quantitative analysis of emission rates; and (b) Greater emission from the pheromone gland when relatively high linear velocity air was drawn over the pheromone gland. The latter point was also documented by Sower et al. [22], and also hypothesized by Kuenen and Hicks [20], who observed high emission rates when a megabore column was placed within < 1 mm of the pheromone gland, and 25 ml/min air flow was drawn through a 3.9 ml glass cylinder. In our design, the funnel would significantly reduce the linear velocity over the gland, as air was drawn from a large volume of air around the gland. In addition, because we linked collection duration to calling duration, our collection per unit time may be higher than in studies that collected continuously from females without regard to calling and resting bouts.

We detected the same 11 components in the collections of volatiles from calling females as in gland extracts (Fig 2). Overall, the ratios of these components from each source (collections of volatiles and gland extracts) were consistent with previous studies, but with some minor differences. The alcohols were highly represented in the gland extracts, followed by the acetates and 16-carbon aldehydes (Fig 2B). *Z*11-16:Ald and *Z*7+*Z*9-16:Ald were the predominant components in the gland volatiles (76.1% and 9.6%, respectively). Except for the 14-carbon aldehydes, the differences in the gland vs. volatile representation of each compound were statistically significant (paired Student’s *t*-test, p < 0.05). The 16-carbon aldehydes were relatively more abundant in the collections of volatiles than in extracts, whereas the alcohols and acetate esters were relatively more represented in the gland extracts than in collections of volatiles (paired Student’s *t*-test, p < 0.05) (Fig 2C). The most remarkable differences in the relative amounts of volatiles and gland extracts were in *Z*11-16:Ald and *Z*11-16:OH, the most abundant components in the gland. *Z*11-16:Ald was more represented in the collections of volatiles, and while *Z*11-16:OH was a prominent component in the gland extracts, it was a minor component in the trapped volatiles. *Z*11-16:OH serves a dual function not only as a pheromone component, but also as precursor of the major sex pheromone component, *Z*11-16:Ald [23–25]

The representation of the major *Z*11-aldehyde, acetate and alcohol in volatile emissions differed substantially between our study and two previous reports. *Z*11-16:Ald constituted 76.2% of the total volatiles in our study, with *Z*11-16:OAc and *Z*11-16:OH at 3.0% and 1.0%, respectively (Fig 2C). The respective percentages in Heath et al. [10] were 53.9%, 7.2% and 3.6% and in Lievers and Groot [14] 59.0%, 2.4% and 0.8%.

## General discussion

There are many situations that require collections of volatiles from individual insects or flowers because individual phenotypes may then be associated with other traits, gene regulation, functional analysis (eg, RNAi) and other approaches that may be masked by a group. The open tubular collection system of volatiles using short sections of megabore column has several notable advantages over typical polymeric adsorbents. First, it requires much smaller volumes of solvent to recover analytes from the traps (see also [20]), enabling micro chemical derivatization with minimal loss of sample components. This feature also facilitates better quantitative analysis with lower limits of detection [26]. In this regard, it is comparable to the system described by Grob and Zürcher [27] using an adsorbent trap that is extracted with a small volume of solvent that is moved up and down through the adsorbent. Second, the megabore column can be cleaned more efficiently, either with solvents, or by thermal desorption in a GC oven by either connecting it to the inlet or to another column with a press-fit connector. Third, the availability of various stationary phases and various film thicknesses makes this a highly adaptable technique that can be optimized for each unique application based on the polarity, volatility, size, mass and other characteristics of the target volatiles. Together, and in combination with TMS derivatization of alcohols and selective and sensitive detection by SIM with GC-CI-MS, this procedure also enables reliable identification and quantification of trace polar analytes on non-polar columns.

Recently, a PDMS-coated fused silica optical fiber was used to collect compounds from the gland by rubbing the gland surface with the fiber for several minutes (ie, direct-contact SPME). The adsorbed materials were extracted with hexane and subjected to GC-FID analysis [14]. The ratios of the pheromone components quantified by this method and polymer adsorbent-based collections of volatiles were similar for 16-carbon aldehydes, but differed substantially for 16-carbon acetates and alcohols as well as 14-carbon aldehydes [14]. This method samples compounds from the outer surface of the pheromone gland, and therefore appears to represent an interface between stored and emitted compounds. The megabore column method can be integrated with this method and gland extractions to inform a better understanding of the dynamics of pheromone production and emission.

We also note two important caveats with this system. In an open system, such as we used in this study, the efficiency of the megabore collection procedure degrades rapidly with increasing distance from the emission source. The silanized glass funnel effectively mitigated this shortcoming with *H*. *subflexa*, but its positioning can be challenging with more mobile insects. Moreover, this approach has limitations in a closed flow-through system, where the emission source in contained in an apparatus. The relatively low flow rate that can be implemented with a short megabore column becomes problematic as the volume of the apparatus increases. Critical to collections of volatile is the ability to implement multiple air exchanges of the entire apparatus. For example, using a flow rate of 10 ml/min, as in our study, and a typical 1000 ml chamber will require 100 min for a single evacuation of the chamber, a clearly unwise combination. Therefore, this collection system should be implemented in small chambers where the linear velocity and complete air exchanges in the chamber can be maximized at relatively low volumetric flow rates. Kuenen and Hicks [20] effectively did so by collecting from females calling within a small 3.9 ml glass cylinder using a 75 cm megabore column at 25 ml/min.

Finally, we recently applied this technique to collections of volatiles from *Heliothis (= Chloridea) virescens*, whose pheromone gland contains *Z*11-16:OH but the role of this alcohol in male attraction has been controversial. Several earlier studies could not detect *Z*11-16:OH in gland emissions of *H*. *virescens*, and flight-tunnel assays failed to attribute to it any conspecific function. Using the thick film megabore column, coupled with derivatization and GC-CI-SIM-MS as described here, we were able to show that *Z*11-16:OH was emitted from the sex pheromone gland of calling females, and field trapping experiments and behavioral observations demonstrated that males were attracted and trap catch increased when *Z*11-16:OH was added to various pheromone blends [28]. This approach enabled us to conclude that *Z*11-16:OH is a component of the sex pheromone of *H*. *virescens* females.

## Supporting information

S1 Table Supporting data. Includes data in support of the figures.
(XLSX)

## References

[1] Wyatt TD. Pheromones and Animal Behaviour: Communication by Smell and Taste. Cambridge: Cambridge University Press; 2003.

[2] The Pherobase: Database of insect pheromones and semiochemicals [Internet]. http://www.pherobase.com. 2018. Available from: http://www.pherobase.com.

[3] Percy-Cunningham JE, MacDonald JA. Biology and ultrastructure of sex pheromone-producing glands In: Prestwich GD, Blomquist GJ, editors. Pheromone biochemistry. Orlando, Fl: Academic Press, Inc.; 1987 p. 27–69.

[4] Allison JD, Cardé RT. Pheromones: Reproductive isolation and evolution in moths In: Allison JD, Cardé RT, editors. Pheromone Communication in Moths: Evolution, Behavior, and Application. Oakland, California: University of California Press; 2016 p. 11–23.

[5] Roelofs WL. Reminiscence of the early days In: Allison JD, Cardé RT, editors. Pheromone Communication in Moths: Evolution, Behavior, and Application. Oakland, California: University of California Press; 2016 p. 3–9.

[6] Allison JD, Cardé RT, editors. Pheromone communication in moths: evolution, behavior, and application. Oakland, California: University of California Press; 2016.

[7] McElvare RR. Validity of the species *Heliothis subflexa* (Gn.) (Lepidoptera). Bull Brooklyn Entomol Soc. 1941;36:29–30.

[8] Teal PEA, Heath RR, Tumlinson JH, McLaughlin JR. Identification of a sex pheromone of *Heliothis subflexa* (Gn) (Lepidoptera, Noctuidae) and field trapping studies using different blends of components. J Chem Ecol. 1981;7(6):1011–22. 10.1007/BF00987623 24420826

[9] Klun JA, Leonhardt BA, Lopez JD, Lachance LE. Female *Heliothis subflexa* (Lepidoptera, Noctuidae) sex pheromone—chemistry and congeneric comparisons. Envir Entomol. 1982;11(5):1084–90. 10.1093/ee/11.5.1084 PubMed PMID: WOS:A1982PM19600022.

[10] Heath RR, McLaughlin JR, Proshold F, Teal PEA. Periodicity of female sex pheromone titer and release in *Heliothis subflexa* and *H*. *virescens* (Lepidoptera, Noctuidae). Ann Entomol Soc Am. 1991;84(2):182–9. 10.1093/aesa/84.2.182 PubMed PMID: ISI:A1991FC55300008.

[11] Groot AT, Santangelo RG, Ricci E, Brownie C, Gould F, Schal C. Differential attraction of *Heliothis subflexa* males to synthetic pheromone lures in eastern US and western Mexico. J Chem Ecol. 2007;33(2):353–68. 10.1007/s10886-006-9233-6 PubMed PMID: ISI:000243557500012. 17200888

[12] Heath RR, Mitchell ER, Tovar JC. Effect of release rate and ratio of (Z)-11-hexadecen-1-ol from synthetic pheromone blends on trap capture of *Heliothis subflexa* (Lepidoptera, Noctuidae). J Chem Ecol. 1990;16(4):1259–68. 10.1007/BF01021024 PubMed PMID: WOS:A1990DA66900018. 24263725

[13] Vickers NJ. Defining a synthetic pheromone blend attractive to male *Heliothis subflexa* under wind tunnel conditions. J Chem Ecol. 2002;28(6):1255–67. 10.1023/a:1016242019571 12184401

[14] Lievers R, Groot AT. Disposable polydimethylsiloxane (PDMS)-coated fused silica optical fibers for sampling pheromones of moths. Plos One. 2016;11(8). 10.1371/journal.pone.0161138 PubMed PMID: WOS:000381487600063. 27533064 PMC4988701

[15] Heath RR, Manukian A. Development and evaluation of systems to collect volatile semiochemicals from insects and plants using a charcoal-infused medium for air purification. J Chem Ecol. 1992;18(7):1209–26. 10.1007/BF00980075 PubMed PMID: WOS:A1992JD59000022. 24254160

[16] Nongonierma A, Cayot P, Le Quere JL, Springett M, Voilley A. Mechanisms of extraction of aroma compounds from foods, using adsorbents. Effect of various parameters. Food Rev Int. 2006;22(1):51–94. 10.1080/87559120500379951 PubMed PMID: WOS:000234545000004.

[17] Qualley AV, Dudareva N. Metabolomics of plant volatiles. Methods Mol Biol. 2009;553:329–43. 10.1007/978-1-60327-563-7_17 PubMed PMID: WOS:000271177400017. 19588114

[18] Nojima S, Apperson CS, Schal C. A simple, convenient, and efficient preparative GC system that uses a short megabore capillary column as a trap. J Chem Ecol. 2008;34(3):418–28. 10.1007/s10886-008-9437-z PubMed PMID: WOS:000253975100015. 18297362

[19] Nojima S, Kiemle DJ, Webster FX, Apperson CS, Schal C. Nanogram-scale preparation and NMR analysis for mass-limited small volatile compounds. Plos One. 2011;6(3):7 10.1371/journal.pone.0018178 PubMed PMID: WOS:000289053800025. 21464906 PMC3065492

[20] Kuenen LPS, Hicks MN. Gas chromatography column as an ambient-temperature volatile trap. Entomo Exp Appl. 2015;154(1):35–44. 10.1111/eea.12253 PubMed PMID: WOS:000346487700005.

[21] Kuenen LPS, Siegel JP. Measure your depta release ratios: pheromone release ratio variability affected by rubber septa and solvent. J Chem Ecol. 2015;41(3):303–10. 10.1007/s10886-015-0557-y PubMed PMID: WOS:000353397400009. 25801328

[22] Sower LL, Shorey HH, Gaston LK. Sex pheromones of noctuid moths. XXV. Effects of temperature and photoperiod on circadian rhythms of sex pheromone release by females of *Trichoplusia ni* (Lepidoptera, Noctuidae). Ann Entomol Soc Am. 1971;64(2):488–92. 10.1093/aesa/64.2.488 PubMed PMID: WOS:A1971I770900037.

[23] Teal PEA, Tumlinson JH. Terminal steps in pheromone biosynthesis by *Heliothis virescens* and *Heliothis zea*. J Chem Ecol. 1986;12(2):353–66. 10.1007/BF01020561 PubMed PMID: ISI:A1986A684000004. 24306785

[24] Morse D, Meighen E. Pheromone biosynthesis: enzymatic studies in Lepidopter a. In: Prestwich GD, Blomquist GJ, editors. Pheromone biochemistry. New York: Academic Press; 1987 p. 212–5.

[25] Choi MY, Groot A, Jurenka RA. Pheromone biosynthetic pathways in the moths *Heliothis subflexa* and *Heliothis virescens*. Arch Insect Biochem Physiol. 2005;59(2):53–8. 10.1002/arch.20051 PubMed PMID: ISI:000229458900001. 15898118

[26] Attygalle AB, Morgan ED. Versatile microreactor and extractor. Anal Chem. 1986;58(14):3054–8. 10.1021/ac00127a034

[27] Grob K, Zürcher F. Stripping of trace organic substances from water. Equipment and procedure. J Chromatogr. 1976;117:285–94. 10.1016/0021-9673(76)80005-2

[28] Groot AT, Nojima S, Heath JJ, Ammagarahalli B, van Wijk M, Classen A, et al Alcohol contributes to attraction of *Heliothis* (= *Chloridea*) *virescens* males to females. J Chem Ecol. 2018;(in press). 10.1007/s10886-018-0995-4 30039209

